# Organic–Inorganic Double-Gel System Thermally Insulating and Hydrophobic Polyimide Aerogel

**DOI:** 10.3390/polym14142818

**Published:** 2022-07-11

**Authors:** Liyao Xiong, Weijie Zheng, Shenglong Cao, Yuying Zheng

**Affiliations:** College of Materials Science and Engineering, Fuzhou University, Fuzhou 350116, China; o5cookie@163.com (L.X.); 13290987197@163.com (W.Z.); csl239230@163.com (S.C.)

**Keywords:** thermal insulation, hydrophobic, thermal stability, aerogel, polyimide

## Abstract

Aerogel materials are used in various fields, but there is a shortage of aerogel materials with an excellent combination of mechanical properties, thermal stability, and easy preparation. In this study, polyimide aerogel materials with superior mechanical properties, thermal stability, and low thermal conductivity were prepared by forming a double-gel system in the liquid phase. The amino-modified gel, prepared by coating SiO_2_ nano-microspheres with GO through a modified sol-gel method (SiO_2_@GO-NH_2_), was subsequently homogeneously dispersed with PAA wet gel in water to form a double-gel system. The construction of a double-gel system enabled the PI aerogel to shape a unique honeycomb porous structure and a multi-layered interface of PI/SiO_2_/GO. The final obtained PI aerogel possessed effective thermal conductivity (0.0309 W/m·K) and a high specific modulus (46.19 m^2^/s^2^). In addition, the high thermal stability (543.80 °C in Ar atmosphere) and the ability to retain properties under heat treatment proved its durability in high thermal environments. The hydrophobicity (131.55°) proves its resistance to water from the environment. The excellent performance of this PI aerogel and its durability in thermal working environments make it possible to be applied in varied industrial and research fields, such as construction and energy, where heat and thermal insulation are required.

## 1. Introduction

The overconsumption of energy is becoming a critical issue, based on engineering, environmental, and economic challenges in modern industries [1,2,3]. Pursuing a novel material with high performance and energy efficiency is a forefront research topic in terms of industrial applications [4,5,6,7]. Aerogel, as an impressive commercial energy material, plays a crucial role in the thermal insulation field due to its high strength and low density, which can be subdivided into inorganic and organic aerogels. Inorganic aerogels, especially silica-derived aerogels, have emerged as representatives of the heat insulation field because of their highlighted inherent textural properties, and, more importantly, unprecedented thermal stability and multifunctional use [8,9,10,11,12,13]. Despite the promising performance of inorganic aerogels, the obvious intrinsic drawbacks, such as hygroscopicity, brittleness, and lack of compressive modulus, inhibit their applications [14]. Compared with inorganic aerogels, organic aerogels, which have tunable structural properties, are not stable in thermal conditions [15,16,17,18,19]. Therefore, developing organic–inorganic aerogels is imperative to achieve the desired performance. Additionally, designing an effective combination strategy represents a critical first step in the establishment of a bridge between inorganic and organic aerogels [14,20,21].

Many strategies have been achieved in the development of high-strength bonding of organic–inorganic aerogels so far. The functional modification of aerogels by introducing nano-fillers has gained significant research attention [22,23,24,25]. In early studies, Wang et al. [26] built blocks of cellulose nanofibers and ZrP/RGO nanosheets, which provided an aerogel with ultralow thermal conductivity (0.018 W/m·K) and high Young’s modulus (194 kPa). Moreover, Zhu et al. [27] created a modified hydroxyapatite/chitosan composite aerogel with good mechanical properties and thermal insulation. Additionally, recent works have exhibited an appealing tendency to form a mutually cross-linked network structure by using a principal skeleton, e.g., silica [20], polyacrylonitrile [28], aramid nanofiber [29], and sodium alginate skeleton [30], etc. However, despite the great success of the strategies mentioned above, the industrial application practicability is still restricted due to the complexity of the process.

In recent years, many attempts have been made to achieve dehydration of wet gel, including regular drying, supercritical CO_2,_ and freeze-drying, etc. Among them, supercritical CO_2_ is considered to be a better candidate in the conversion from wet gel to aerogel, which could deliver an excellent microscopic pore structure. In early studies, Zhang et al. [31] prepared impressive low-density (~0.2 g/cm^3^) aerogels using supercritical drying. In parallel, Ana Iglesias-Mejuto and her co-workers [32] verified the compatibility of 3D printing and supercritical CO_2_ drying in the field of aerogels. Despite their promising performance, poor mechanical properties, lack of thermal stability, and complex processes remain their fatal flaws [5]. Accordingly, there is an urgent demand for a modified method to address the aforementioned issues by producing better performance.

Herein, we introduce a “double-gel” method in which SiO_2_@GO-NH_2_ gels were conveniently and effectively constructed using the sol-gel method, and dispersed in an aqueous system to form a double-gel system with poly amic acid (PAA) organic wet gels. The aqueous system allowed the two phases to be uniformly dispersed and mixed, while the amino modification improved the compatibility between both gels. After freeze-drying and heat treatment, the wet gels turn into aerogels during PAA and transform into one of the most thermally stable polymeric materials known, polyimide (PI). The multilayered interfacial structure and the compatible dispersion of the two phases bring superior mechanical properties, thermal insulation, thermal stability, thermal durability, and hydrophobicity to aerogels.

## 2. Materials and Methods

### 2.1. Materials

4,4′-diaminodiphenyl ether (ODA), pyromellitic dianhydride (PMDA), tetraethyl orthosilicate (TEOS), ammonium hydroxide solution (25%), and N,N-dimethylacetamide (DMAc) were supplied by Shanghai Macklin Biochemical Co., Ltd. (Shanghai, China). Triethylamine (TEA) and (3-aminopropyl) triethoxysilane (3ATPES) were purchased from Aladdin Reagent Co., Ltd. (Shanghai, China). Ethanol, potassium permanganate, sulfuric acid (98%), and hydrochloric acid (36–38%) were purchased from Sinopharm Chemical Reagent Co., Ltd. (Shanghai, China). Flake graphite was provided by Shanghai Jingchun Reagent Co., Ltd. (Shanghai, China). All the reagents mentioned above were analytical grade, unless otherwise indicated.

### 2.2. Preparation of Amino-Modified SiO_2_@GO (SiO_2_@GO-NH_2_) Wet Gel by Sol-Gel Method

Graphene oxide (GO) was prepared using a modified Hummers’ method [33].

SiO_2_@GO -NH_2_ wet gels were prepared using the sol-gel method. Specifically, 25 mg of GO was added to 75 mL of 20 vol% TEOS ethanol solution, ultrasonically treated for 30 min, which ensured the system was well dispersed. Afterward, 10.5 mL of 0.1 M hydrochloric acid was added to acidify the system and was then ultrasonically treated for 5 min to fully hydrolyze the TEOS. A total of 4.5 mL of 10.79 wt.% 3ATPES ethanol solution was titrated slowly with slow mechanical stirring to gel the system and stirred for 24 h. The SiO_2_@GO-NH_2_ wet gel was then obtained.

### 2.3. Preparation of Polyamic Acid (PAA) Precursors and Wet Gel

Using an ice bath, 5.2630 g of ODA was dissolved in 55 mL of DMAc at 0 °C. After stirring for 5 min, 4.7369 g of PMDA was slowly added to the system in batches. The molar ratio of PMDA-to-ODA was 50:51 to control the length of polymer chains. The product was slowly poured into DI water at 0 °C and washed four times with DI water and freeze-dried at −65°C to obtain the PAA precursor. The ether bonding of ODA and the non-coplanar PAA chains make the PAA chains more soluble in the chosen solvent (water) and facilitate hydrogen bonding interactions with the inorganic doped material [34,35]. The PAA precursor was mixed with water and TEA in a mass ratio of 1:10:0.5 at room temperature and stirred at high speed for 30 min to obtain the PAA wet gel [36].

### 2.4. Preparation of Polyimide (PI) Aerogels

The SiO_2_@GO-NH_2_ wet gel was dispersed in 7.5 g of water by high-speed stirring and ultrasound. This was followed by continuous stirring in an ice bath at 0 °C using a slower stirring speed, while adding a total of 11.5 g of PAA wet gel in five batches. Stirring was continued for 4 h until the system was homogeneous. The obtained organic–inorganic dual system wet gels were poured into molds and frozen in a cold trap at −65 °C for 4 h, before being freeze-dried for 48 h. The molds were pre-cooled in the cold trap in advance, with the lowest cold trap temperature chosen to reduce the size of ice crystals in the gel system. The obtained aerogels were heat-treated at 80 °C for 30 min, 110 °C for 30 min, 195 °C for 120 min, and 290 °C for 60 min under nitrogen protection to obtain PI aerogels and numbered PI-1 (1 represents the addition of 1 g SiO_2_@GO-NH_2_). Similarly, a series of PI aerogels were prepared with different ratios of SiO_2_@GO-NH_2_ wet gels to study the effect of doping amount on the aerogel properties; the prepared PI aerogels were named PI-0, PI-0.5, PI-1.5, and PI-2. The PI aerogels used for mechanical and thermal property tests were prepared using specific molds. The diameter of the obtained PI aerogel was 35 ± 0.5 mm and the height was 10 ± 0.2 mm.

### 2.5. Characterization

The Nicolet 570 FTIR spectrometer (Thermo Fisher Scientific, Agawam, MA, USA) was used to obtain FTIR spectra of SiO_2_@GO-NH_2_. Microstructure pictures and EDX maps of freeze-dried SiO_2_@GO-NH_2_ wet gels and PI aerogels were taken with a SUPRA 55 thermal field emission scanning electron microscope (Carl Zeiss, Berlin, Germany) (SEM). ATR-FTIR spectra of PI aerogels were obtained with a Nicolet iS50 Fourier Transform Infrared Spectrometer (Thermo Fisher Scientific, MA, USA). XRD data of PI aerogels were provided by using an ULTIMA III X-ray diffractometer (Nippon Rigaku, Tokyo, Japan). The DXR2Xi Confocal Laser Raman Spectrometer (Thermo Fisher Scientific, MA, USA) provided Raman spectra of PI-0, PI-0.5, and PI-1.5 samples. The photoelectron spectra of PI-0 and PI-1.5 aerogels were measured using the ESCALAB 250 X-ray photoelectron spectrometer (Agawam, VG, USA). The ^13^C NMR data of PI-1.5 aerogel were acquired using an AVANCE Ⅲ 500 Nuclear Magnetic Resonance Spectrometer (Bruker, Fällanden, Switzerland). The stress–strain curves of the aerogel samples were obtained using an INSTRON-1185 (INSTRON, Norwood, MA, USA). The thermal conductivity of the aerogel samples was investigated using a DZDR-S thermal conductivity meter (Dazhan Mechatronics, Singapore). The infrared photographs of PI-0 and PI-1.5 placed on the heating stage were taken using a DS-2TPH10-3AUF infrared camera (HIKVISION, Hangzhou Hikvision Digital Technology Co., Ltd., Hangzhou, China). The thermogravimetric data of each group of PI aerogels were tested using a STA449C/6/G simultaneous thermal analyzer (NETZSCH, Westendstr, Germany). The water contact angle of each group of PI aerogel samples was measured with a SL200A/B dynamic/static contact angle meter (Shanghai Solon, Shanghai, China).

## 3. Results and Discussion

### 3.1. Amino-Modified SiO_2_@GO (SiO_2_@GO-NH_2_) Wet Gel

**Figure 1:**
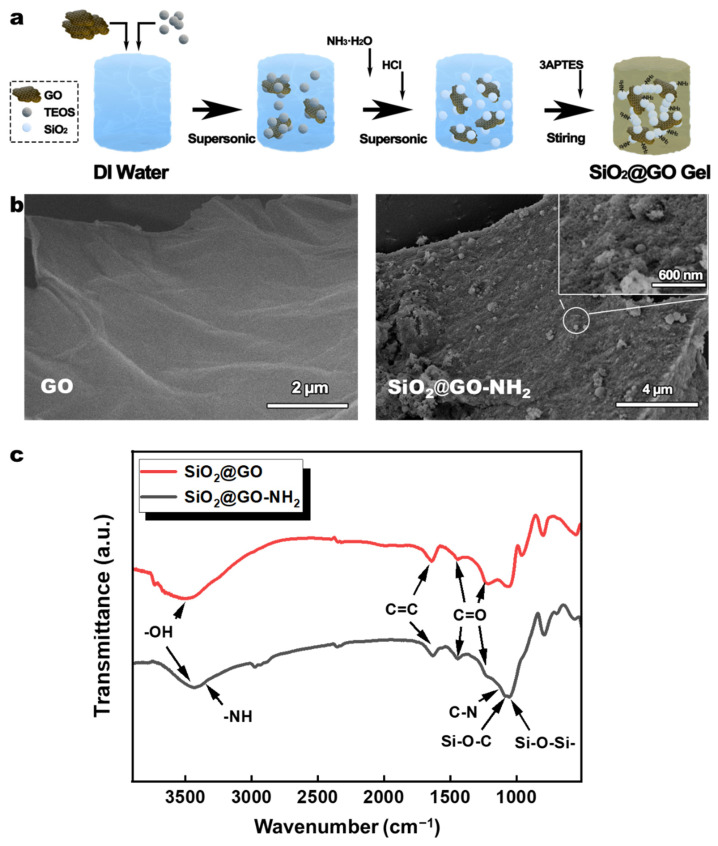
(**a**) Preparation process of SiO_2_@GO-NH_2_ wet gel. (**b**) SEM images of GO and SiO_2_@GO-NH_2_. (**c**) FT-IR spectra of SiO_2_@GO and SiO_2_@GO-NH_2_.

**Figure 2:**
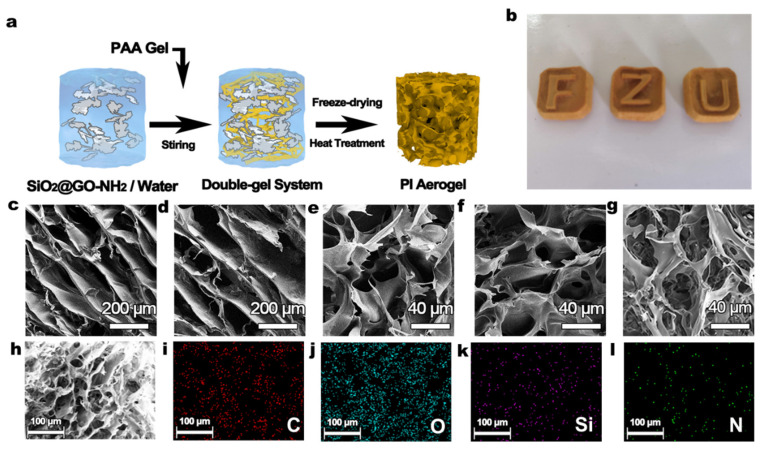
(**a**) Preparation process of double-gel PI aerogel. (**b**) A set of PI aerogels was prepared using custom molds, demonstrating the scalability of the preparation process. (**c**–**g**) SEM images of PI-0, PI-0.5, PI-1, PI-1.5, and PI-2. (**h**) Another SEM image and (**i**–**l**) EDS maps of PI-1.5 in the same area.

The preparation process of SiO_2_@GO-NH_2_ wet gel is shown in Figure 1a. The SEM image in Figure 2b shows uniform accumulation of silica particles on the graphene sheets, indicating that the silica in the wet gel system is grown diffusively as nano-spheres with graphene as the base. FT-IR spectra of SiO_2_@GO-NH_2_ gels prepared by the above experimental scheme, and general SiO_2_@GO gels with the replacement of 3APTES with ammonia, are shown in Figure 1c. The peaks of both spectra contain -OH (3400 cm^−1^), C=C (1630 cm^−1^), and C=O (1350 and 1230 cm^−1^) from GO, Si-O-Si- (1090 cm^−1^), SiO_2_, and Si-O-C (1130 cm^−1^), which represents the interaction between SiO_2_ and GO. In addition to the peaks shared in SiO_2_@GO-NH_2_, -NH_2_ (3260 cm^−1^) and C-N (1220 cm^−1^) are also present. Such data indicate that 3ATPES underwent hydrolysis and co-growth with SiO_2_ nano-spheres during the sol-gel process in the alcohol–water system. This co-growth process also serves to amino-modify the silica nano-spheres by functionalization through 3APTES [37].

### 3.2. Preparation of PI Aerogel with Inorganic–Organic Double-Gel System

The preparation process of the PI aerogel is shown in Figure 2a. Specifically, this was to form a uniformly dispersed SiO_2_@GO-NH_2_ system from controlled quality SiO_2_@GO-NH_2_ gel in water, using high-speed stirring and ultrasonic treatment. Figure 2b shows the excellent processing properties of this aerogel preparation method, which can form finer and special structures with different molds. Figure 2c–h show typical SEM photographs of various PI aerogels. PI-1 to PI-2 formed a unique honeycomb, showing the limiting effect of the SiO_2_@GO-NH_2_ inorganic gel network on synergistic growth of the ice crystal/PAA linkage [38]. For comparison, PI-0 and PI-0.5 displayed a larger needle-like pore structure due to the spontaneous growth of polymer chains in the direction of the ice crystal growth edge during the freezing process. Typically, SiO_2_@GO-NH_2_ wet gel introduced in the PI-0.5 system is not enough to form an inorganic gel network, making the microstructure more similar to that of PI-0. The large pore size exhibited in PI-0 and PI-0.5 proves the difficulty of controlling the microscopic scale of polyimide aerogels prepared using the general freeze-drying method. In particular, the pores of PI-2 showed a lot of merging and fragmentation. The abnormal properties of PI-2 might be caused by the excessive addition of SiO_2_@GO-NH_2_ providing an overdone limiting effect, making it more difficult to form a stable honeycomb structure [39].

The elemental distribution on the microscopic scale of PI-1.5 was measured and analyzed using EDS. The results showed uniformity of the elements, while the microstructure of the original aerogel can still be observed in the images of each element, especially Si. The results observed by EDS demonstrate the homogeneity of the double-gel system, which means that the PI and the SiO_2_@GO-NH_2_ form the aerogel honeycomb structure on the nanoscale together.

### 3.3. Chemical Structure of PI Aerogel

**Figure 3:**
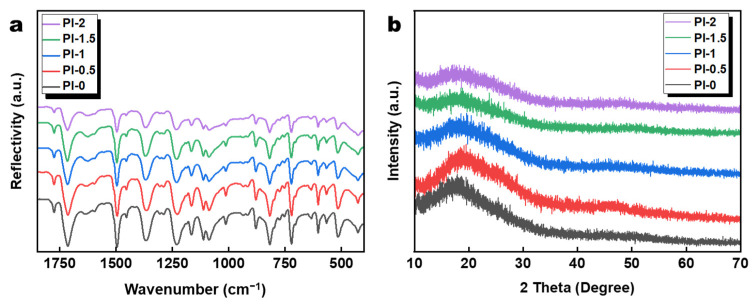
(**a**) FTIR-ATR spectra and (**b**) X-ray diffraction patterns of PI-0, PI-0.5, PI-1, PI-1.5, and PI-2.

The ATR-FTIR infrared spectra of the aerogel materials are shown in Figure 3a. The strong characteristic peaks at 1774 and 1710 cm^−1^ occurring on all spectra are attributed to the symmetric and asymmetric stretching of the C=O group of the imide double bond. The peak at 1375 cm^−1^ is attributed to the C-N stretching vibration, and a C=C stretching vibration at 1495 cm^−1^ was also observed. It can be concluded that the doping of SiO_2_@GO-NH_2_ and the double-gel network structure do not affect the chemical process of PI formation by imidization of PAA [40,41]. Figure 3b shows typical XRD data for each PI aerogel. All PI aerogels display characteristic polyimide amorphous structure peaks, but PI-0 and PI-0.5 have higher peak heights compared to other samples, which is related to the oriented structure formed during the aerogel and preparation process [42]. This also proves that the introduction of the SiO_2_@GO-NH_2_ gel changes the overall structure of polyimide aerogels.

**Figure 4:**
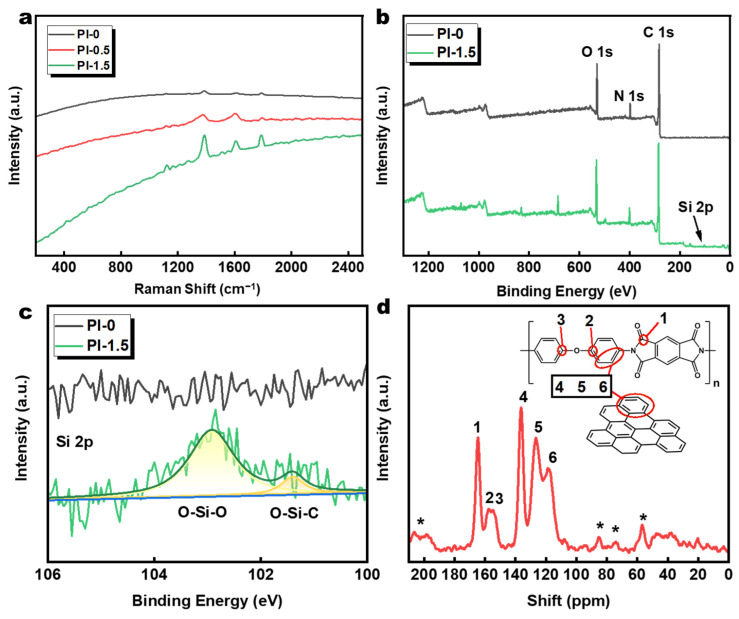
(**a**) Raman spectra of PI-0, PI-0.5, and PI-1.5. (**b**) XPS and (**c**) Si 2p spectra of PI-0 and PI-1.5. (**d**) Solid-state ^13^C NMR spectra of the PI-1.5; * indicates peaks from spinning sidebands.

Figure 4a shows the Raman spectra of PI-0, PI-0.5, and PI-1.5. All three spectra exhibited bright fluorescent signals, and the spectral bands around 1340 cm^−1^ can be attributed to the C-N stretching vibration, around 1590 cm^−1^ to the ring vibration of PI, and around 1740 cm^−1^ to the stretching vibration of C=O from PI and GO [43,44]. This may be caused by the convergence of the D-band of rGO (1330 cm^−1^) and C-N stretching vibration (1340 cm^−1^) with each other, the overlap of the G-band of rGO (1560 cm^−1^) and the ring vibration of PI (1590 cm^−1^), and the enhancement of the peak signal with an increase in the SiO_2_@GO-NH_2_ [45]. This evidence demonstrates the close association between PI chains and SiO_2_@GO-NH_2_ within aerogels.

To further verify the chemical bond structure and elemental composition of the aerogel material, XPS was used to analyze the PI-0 and PI-1.5 materials. The peaks of Si 2p can be deconvoluted into two groups of sub-bands, assigned to O-Si-O (102.9 eV) and O-Si-C (101.2 eV), representing the amino-modification by 3ATPES [37,46,47,48].

The solid-state ^13^C NMR further reveals the chemical structure described by the previous characterizations. The peaks at 118, 123, and 136 ppm belong to graphene and aromatic carbon peaks on the PI chains [49]. The peak at 165 ppm belongs to the carbonyl carbon [50]. The peaks at 155 and 158 ppm, except for the observed double peaks, belong to aromatic carbon forming ether bonds on the PI chains, which are slightly shifted due to their chemical environment [51]. The peaks at 155 and 158 ppm belong to aromatic carbons forming ether bonds in the PI chains.

### 3.4. Properties of PI Aerogel

**Figure 5:**
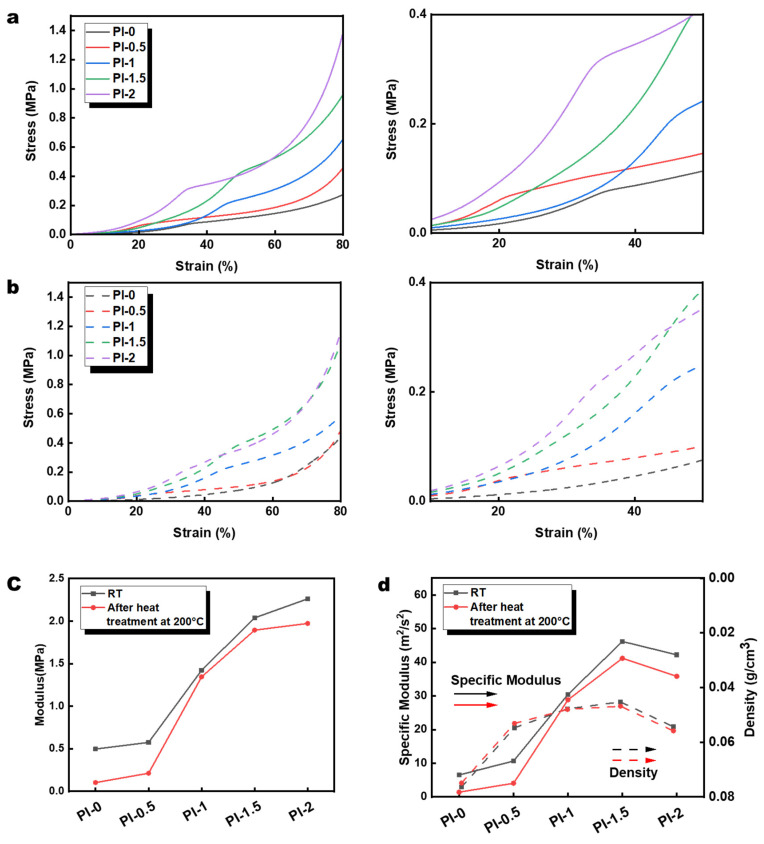
(**a**,**b**) The stress–strain curves, (**c**) Young’s moduli, (**d**) specific moduli, and densities of PI-0, PI-0.5, PI-1, PI-1.5, and PI-2.

In practical applications, mechanical properties are often the key element in measuring the performance of aerogels. The densities of aerogels were acquired by averaging the results of three measurements of a 10 mm × 10 mm × 10 mm sized PI aerogel. In this way, we further obtained the specific moduli data of PI aerogels. Although it is generally believed that limited SiO_2_ nanoparticles can improve the mechanical properties of polymeric materials to a certain extent, an excess can also lead to a decrease in mechanical properties [52,53]. This is because the addition of more SiO_2_ leads to agglomeration of nanoparticles due to van der Waals forces and electrostatic adsorption, resulting in stress concentration between the SiO_2_ and the polymer interface [54]. Although a large amount of SiO_2_@GO-NH_2_ was added into the system during the preparation of the PI aerogel in this study, the specific modulus of the original PI-1.5 sample still reached a relatively superior 46.19 m^2^/s^2^ while maintaining a low density (0.0441 g/cm^3^). There are several reasons for this: (1) the high dispersion of SiO_2_@GO-NH_2_, (2) amino groups led to SiO_2_@GO-NH_2_ providing crosslinking sites and enhancing material compatibility, (3) the loading effect played by GO, and (4) the improvement in the structural properties of the given substrate by the silica-containing network [39,55]. As shown in Figure 5a, PI-0 and PI-0.5 samples have similar stress–strain curves, probably due to the similarity in microstructure. With the improvement in microstructure, the Young’s moduli of the PI-1 and PI-1.5 samples are greatly enhanced. Interestingly, the two groups of aerogels are still maintained in the linear elastic region when strain exceeds 40%. Then, there is a sharp increase in the platform strain behavior and the densification state. This can be attributed to the energy dissipation effect of the cross honeycomb material and graphene with good toughness values [56]. However, the linear elastic region of PI-2 produces a certain decrease with damage to the microstructure and stress concentration, caused by excessive doping.

As a representative of heat-resistant polymers, PI aerogels should maintain good performance even after the influence of high temperatures. The PI aerogel was heat-treated at a constant temperature of 200 °C in the air for 24 h to investigate the property retention of PI aerogel after long-time heat treatment. The stress–strain curves of the heat-treated aerogels shown in Figure 5b, and the Young’s moduli, specific moduli, and density data of the two sets of aerogels were statistically analyzed accordingly and are summarized in Figure 5c,d. It is not difficult to see that the moduli and specific moduli of polyimide aerogels decreased after heat treatment, created by the relaxation of internal stress and the destruction of the microstructure of polyimide aerogels after heat treatment [57]. However, PI-1.5 still maintains a good Young’s modulus (1.89 MPa) and specific modulus (41.21 m^2^/s^2^), which proves its durability and mechanical property retention under a long-term thermal environment. PI-1.5 produces huge mechanical property improvement compared with PI-1, based on the increase in filler content for polymeric materials, with constant microstructure and uniform dispersion of fillers [58]. Toughening is achieved. The Young’s modulus of PI-2 (2.26 MPa) in PI aerogel preserved at room temperature without heat treatment improves compared with PI-1.5 (2.04 MPa). With an increase in density due to destruction of the microstructure (*ρ*_PI-2_ = 0.0536 g/cm^3^), the specific modulus of PI-2 (42.23 m^2^/s^2^) was shown to decrease. Instead, the Young’s modulus of PI-1.5 and PI-2 (1.97 MPa) became closer after heat treatment. The stress–strain curves showed that the heat-treated PI-2 samples could also be maintained in the linear elastic region at more than 40% of the strain, further indicating the relaxation effect of heat treatment on the concentration of internal stress in the aerogel material.

**Figure 6:**
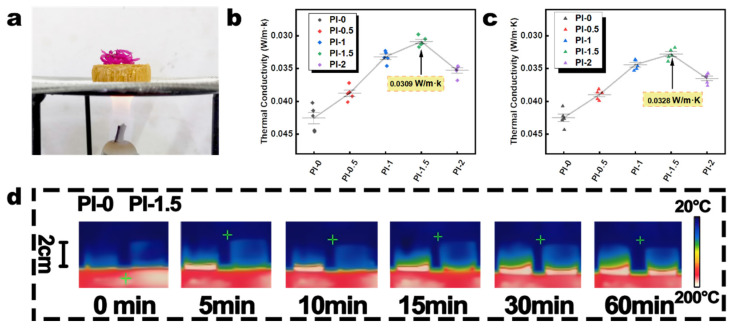
(**a**) PI-1.5 and flowers on the flame of the alcohol burner for 15 min. (**b**,**c**) Thermal conductivity of PI-0, PI-0.5, PI-1, PI-1.5, and PI-2 before and after heat treatment. (**d**) Infrared photo of PI-0 and PI-1.5 placed on a 200 °C heating stage for 60 min.

Thermal insulation is an essential indicator of the energy efficiency of PI aerogels. The thermal resistance analysis of the aerogel material is exhibited in Figure 6. The PI-1.5 aerogel exhibited superior thermal resistance in the original state (0.0309 W/m·K) and after heat treatment (0.0328 W/m·K). The PI-1.5 aerogel exhibits competitive thermal conductivity in comparison with other similar PI aerogel/foam materials (Xue et al. 0.036 W/m·K [39], Zhang et al. 0.0311 W/m·K [51], and Jiang et al. 0.04106 W/m·K [59]). As shown in Figure 6a, the flowers placed on top of PI-1.5 showed no significant dehydration after 15 min of burning using an alcohol burner flame. The introduction of graphene generally leads to an increase in the thermal conductivity of the system, with the wrapping of graphene using SiO_2_ preventing the formation of thermal pathways between graphene, thus eliminating its thermal conductivity [60,61]. The dispersion interval of the thermal resistance test data of the PI-0 sample has a larger error interval compared to that of the other samples, which may be due to the large pore size of the PI-0 sample. Unlike the similarity of mechanical properties, the thermal resistance of PI-0.5 (original: 0.0387 W/m·K; after heat treatment 0.0390 W/m·K) is much better than PI-0 (original: 0.0426 W/m·K; after heat treatment: 0.0425 W/m·K). The uniform dispersion of the inorganic gel phase in the aerogel system forms a PI/SiO_2_/GO multilayer interface, which enhances the thermal resistance (Kapitza thermal resistance) [62]. The thermal resistance of PI-1 (original: 0.0332 W/m·K; after heat treatment: 0.0344 W/m·K) and PI-1.5 increased significantly compared to other aerogels, suggesting that the double-gel system improves the density and microscopic pore structure of the aerogel system. The increased content of SiO_2_@GO-NH_2_ enhanced the Kapitza thermal resistance of the system. As shown in Figure 6d, the infrared images of PI-0 and PI-1.5 are obtained by cutting the original aerogel samples into cylindrical test samples of 2 cm in height and 2 cm in diameter, and then placing them on a 200 °C heating stage for 60 min and photographing them using an infrared camera. PI-1.5 shows much slower thermal diffusion than PI-0, and the thermal resistance is consistent. The superior thermal and mechanical properties make the PI aerogel in this study a possible solution for construction, energy, and other applications [16].

**Figure 7:**
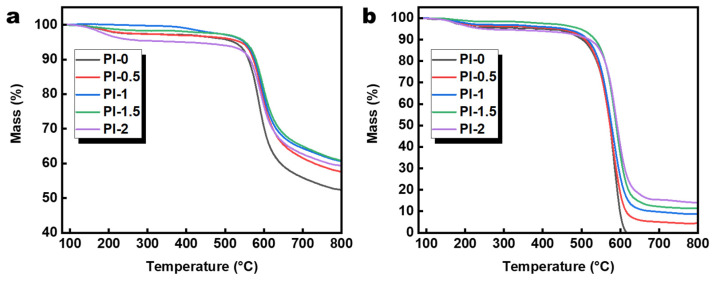
TGA curves of PI-0, PI-0.5, PI-1, PI-1.5, and PI-2 under (**a**) Ar and (**b**) air atmosphere.

The thermal properties of PI aerogels are discussed further by thermogravimetric analysis. The TGA data of PI aerogels were tested and analyzed, and are shown in Figure 7. PI-0 and PI-0.5 showed similar weight loss changes at 0–500 °C and the curves of both almost overlapped. The aerogel microstructure and other factors produce improvement with the addition of SiO_2_@GO-NH_2_, causing decreased weight loss rates of PI-1 and PI-1.5 at 0–500 °C compared to PI-0 and PI-0.5. The decomposition temperatures of PI-0, PI-0.5, PI-1, and PI-1.5 were 522.45, 539.30, 548.88, and 543.80 °C, respectively, when the weight loss reached 5% in the Ar atmosphere. While in the air, they were 384.03, 440.55, 457.22, and 496.03 °C. The improvement in thermal stability demonstrates the strong interaction between organic and inorganic components in the double-gel system [63]. In contrast, the weight loss rate and decomposition temperature (392.12 °C in Ar atmosphere and 248.35 °C in Air atmosphere) of the PI-2 aerogel at 0–500 °C produced a considerable decrease, indicating that the excess SiO_2_@GO-NH_2_ for the disruption of the microstructure of the PI aerogel instead reduced the thermal stability of the aerogel [64].

**Figure 8:**
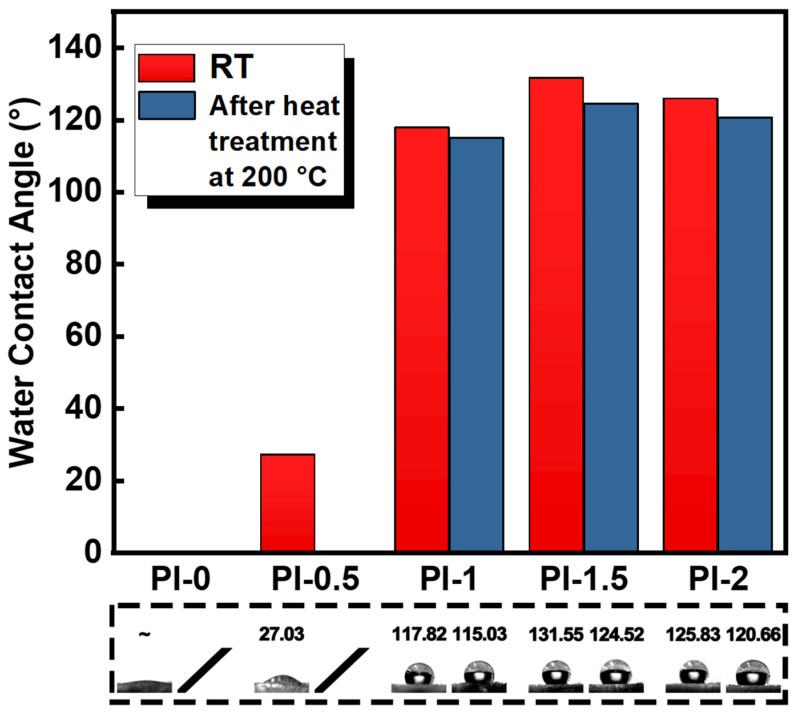
Contact angle data of PI-0, PI-0.5, PI-1, PI-1.5, and PI-2.

If the PI aerogel can absorb moisture from the environment, the thermal insulation property could also reduce. As shown in Figure 8, the hydrophobicity of the PI aerogel was studied by measuring the water contact angle. Due to the capillarity caused by the poor microscopic structure of PI-0, the PI-0 aerogel absorbed all the tested droplets, indicating intrinsic hydrophilicity. The PI-0.5 sample had a water contact angle of 27.03°, indicating that the SiO_2_@GO-NH_2_ gel could provide the hydrophobicity of the PI aerogel. The water contact angle of PI-1 and PI-1.5 produced a tremendous leap. More specifically, the water contact angle of PI-1.5 reached 131.55°, which testifies to its high hydrophobicity. The controlled pore structure of aerogels allows an increase in surface roughness, thus improving the hydrophobicity of the aerogel surface. However, with heat treatment, the water contact angles of the PI aerogels all decreased. The increasing hydrophilicity can be attributed to the introduction of oxygen-containing functional groups or changes in the microstructure [12,65].

## 4. Conclusions

In summary, a double-gel system polyimide aerogel material was discussed in this study. A SiO_2_@GO-NH_2_ amino-modified wet gel was first fabricated via a novel sol-gel method, to obtain a SiO_2_ uniformly coated GO material. Then, the PAA gel and SiO_2_@GO-NH_2_ gel were homogeneously dispersed under the liquid phase, to provide the double-gel system. After a series of subsequent treatments, the prepared PI aerogel had a microscopic porous honeycomb structure and multiple-layer interface, which provided good thermal resistance. The strong compatibility and binding of SiO_2_@GO-NH_2_ with PI also improved the low density (0.0442 g/cm^3^), high specific modulus (46.19 m^2^/s^2^), effective thermal insulation properties (0.0309 W/m·K), high thermal stability (543.80 °C in Ar atmosphere), high hydrophobicity (131.55°), and ability to maintain performance after prolonged exposure to thermal environments. The performance improvement was due to the scientific introduction of SiO_2_@GO-NH_2_ and the construction of the double-gel system. The easy preparation process and superior performance make it suitable for wide applications within various industrial and research fields requiring thermal insulation.

## Data Availability

Not applicable.
